# Whole‐body dose equivalent including neutrons is similar for 6 MV and 15 MV IMRT, VMAT, and 3D conformal radiotherapy

**DOI:** 10.1002/acm2.12543

**Published:** 2019-02-21

**Authors:** Pascal Hauri, Uwe Schneider

**Affiliations:** ^1^ Department of Physics University of Zurich Zurich Switzerland; ^2^ Radiotherapy Hirslanden Hirslanden Medical Center Aarau Switzerland

**Keywords:** radiotherapy, 15 MV vs 6 MV, out‐of‐field dose, neutron dose equivalent

## Abstract

**Purpose:**

This study investigates the difference in whole‐body dose equivalent between 6 and 15 MV image‐guided radiotherapy (IGRT) for the treatment of a rhabdomyosarcoma in the prostate.

**Methods:**

A previously developed model for stray radiation of the primary beam was improved and used to calculate the photon dose and photon energy in the out‐of‐field region for a radiotherapy patient. The dose calculated by the treatment planning system was fused with the model‐calculated out‐of‐field dose, resulting in a whole‐body photon dose distribution. The peripheral neutron dose equivalent was calculated using an analytical model from the literature. A daily cone beam CT dose was added to the neutron and photon dose equivalents. The calculated 3D dose distributions were compared to independent measurements conducted with thermoluminescence dosimeters and an anthropomorphic phantom. The dose contributions from the IGRT treatments of three different techniques applied with two nominal X‐ray energies were compared using dose equivalent volume histograms (DEVHs).

**Results:**

The calculated and measured out‐of‐field whole‐body dose equivalents for the IGRT treatments agreed within (9 *±* 10) % (mean and type A SD). The neutron dose equivalent was a minor contribution to the total out‐of‐field dose up to 50 cm from the isocenter. Further from the isocenter, head leakage was dominating inside the patient body, whereas the neutron dose equivalent contribution was important close to the surface. There were small differences between the whole‐body DEVHs of the 6 and 15 MV treatments applied with the same technique, although the single scatter contributions showed large differences. Independent of the beam energy, the out‐of‐field dose of the volumetric‐modulated arc therapy (VMAT) treatment was significantly lower than the dynamic intensity‐modulated radiation therapy (IMRT) treatment.

**Conclusion:**

The calculated whole‐body dose helped to understand the importance of the dose contributions in different areas of the patient. Regarding radiation protection of the patient for IGRT treatments, the choice of beam energy is not important, whereas the treatment technique has a large influence on the out‐of‐field dose. If the patient is treated with intensity‐modulated beams, VMAT should be used instead of dynamic IMRT in terms of radiation protection of the patient. In general, the developed models for photon and neutron dose equivalent calculation can be used for any patient geometry, tumor location, and linear accelerator.

## 1 INTRODUCTION

Around 10% of long‐term cancer survivors develop a second tumor. Ten percent of these second tumors are induced by the radiation treatment the patient received.^1^ Most second cancers occur at the peripheral region where the dose is greater than 3.0 Gy.^1^ However, Diallo et al.^2^ identified a peak frequency in second malignant neoplasm (including spontaneous cancers) for volumes that received a dose smaller than 2.5 Gy. In external radiation beam therapy, the treated volume receives a high dose while the remaining body is exposed to an unwanted low dose of radiation. Usually, the dose is calculated around the target volume and the out‐of‐field dose is not accurately considered, if at all.^3^ Therefore, whole‐body dose distributions are needed for accurate cancer risk estimates and for optimizing treatment plans by minimizing the cancer risk.

Another motivation for whole‐body dose calculation is the radiation protection of the fetus. Negative effects for a fetus can be substantially minimized if the dose to it is reduced to 100 mGy.^4^ However, practical models to estimate the fetal exposure for intensity‐modulated treatments of pregnant patients do not yet exist.^1^

Takam et al.^5^ presented the current status of out‐of‐field neutron and photon leakage dose in radiotherapy and the associated risk for the patient. Most of their results were based on patient treatments which occurred decades ago. Therefore, studies including novel treatment machines and techniques are urgently needed.^5^

For the same technique applied with different nominal X‐ray energies, the target coverage, conformity, and homogeneity of the treatments are similar.^6^ The choice of nominal X‐ray energy should be based on normal tissue complication probability and on radiation protection issues of the patient. Many studies investigated the difference in the peripheral dose between high (*≥*10 MV) and low nominal X‐ray energy (*<*10 MV).^7^, ^8^, ^9^, ^10^, ^11^ A Monte Carlo (MC) study conducted by Kry et al.,^7^ showed a similar photon out‐of‐field dose for 6 MV compared to 18 MV intensity‐modulated radiation therapy (IMRT) treatments. For the nine organ locations investigated, the simulated neutron doses were typically much lower than the corresponding photon dose. Nevertheless, they warranted an improved neutron dosimetry in order to achieve superior estimates. Ruben et al. 8 measured the components of the out‐of‐field photon dose for 6 and 18 MV treatments. The neutron dose contribution was obtained from published data. They reported that X‐ray energy does not affect the total photon scatter for the same treatment technique. However, the additional neutron dose for 18 MV may have increased total body cancer risk compared to 6 MV IMRT treatments. However, they were not able to draw a firm conclusion. Hälg et al.^12^ used track etch detectors to measure neutron dose equivalent in an anthropomorphic phantom for various treatment modalities. For 15 MV external photon beam treatments, the neutron dose was by factors lower compared to other literature.

With increasing number of treatments using volumetric‐modulated arc therapy (VMAT) and similar dose distributions of VMAT compared to IMRT treatments, the question arises about the difference in the out‐of‐field dose between the two techniques. To our knowledge, there is no study published comparing the peripheral dose (including neutrons) of high‐energy VMAT treatments with IMRT treatments. In the current study, the difference between the dose equivalent of 6 and 15 MV treatments was examined. It is per se not clear that for 15 MV X‐ray nominal beam energy the out‐of‐field dose will be smaller in comparison to 18 MV because of the reduced photoneutron production. Compared to photons, neutrons are a minor part of the total out‐of‐field dose equivalent.^1^ Howell et al. 9 reported a higher effective dose for 15 MV compared to 18 MV 3D‐conformal radiation therapy (3DCRT) treatments.

Also the use of X‐ray imaging modalities can give a substantial dose to the patient.^13^ The choice of treatment technique and indication determines the image modality and therefore, the additional amount of dose to the patient. For patient positioning, the imaging dose is justified by the reduction of the margins around the target. A smaller planning target volume will lead to a sparing of the organs at risk during irradiation. If image‐guided radiotherapy (IGRT) is used, the patient receives an additional dose from X‐ray imaging. By including all contributions of the whole‐body dose for an IGRT treatment, a better understanding in radiation protection of the patient can be achieved.

In the current study, we investigated the whole‐body dose equivalent for 6 and 15 MV IGRT treatments of a rhabdomyosarcoma in the prostate applied with three different techniques (3DCRT, IMRT, and VMAT). The analytically calculated dose distributions were verified with whole‐body dose measurements. The results from the calculation were used to identify differences in the whole‐body dose between the investigated treatments.

## 2 METHODS AND MATERIALS

In this manuscript, the indexes *m*,* c*, and *s* describe quantities which were derived either from measurements, calculations, or MC simulations, respectively. The abbreviation *n* stands for neutrons and *γ* for photons.

### 2.A Whole‐body photon and neutron dose calculation

**Figure 1:**
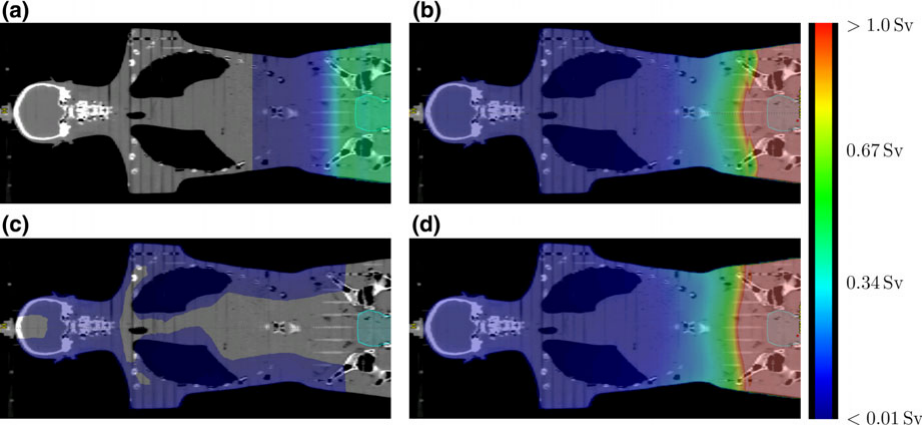
The whole‐body dose equivalent for the 15 MV IMRT treatment with a daily CBCT. The dose equivalent is shown for (a) 23 times a CBCT, (b) photon scatter radiation fused with the treatment planning system calculation, (c) neutrons and (d) the summation of (a)–(c). The Fractionation scheme is presented in Table 1. Furthermore, the outline of the rhabdomyosarcoma in the prostate can be seen.

Dose calculations were performed for an anthropomorphic phantom (Alderson‐Rando, RSD Radiology Support Devices, Long Beach, CA, USA) using a whole‐body grid with a voxel dimension of 0.2 *×* 0.2 *×* 0.5 cm^3^ (see Fig. 1). However, the radiotherapy photon and neutron dose models used in this work are generally applicable to any 3D‐patient data set.

#### 2.A.1 Photon imaging dose

For each treatment fraction, the patient was assumed to be positioned with a full trajectory kV cone beam CT (CBCT) of the pelvis. Hence, we assigned a relatively high imaging dose for the IGRT treatments. The mean absorbed CT dose per Alderson slice was calculated using the average of the thermoluminescence detector (TLD) dose measurements in the corresponding slice. The TLD measurements of the full trajectory pelvis CBCT are reported in Hauri et al.^14^ The absorbed dose per voxel of a CBCT scan was calculated by interpolating the average CBCT dose per Alderson slab along the medial patient axis (MPAX). Hence, the dose was the same for all voxels in a transversal dose‐grid slice [see Fig. 1(a)]. According to Schneider et al.,^15^ the dose of a full rotation CBCT is in a first approximation homogeneous in a transversal slice.

#### 2.A.2 Therapy dose photons and neutrons

A previously developed photon stray dose model for static and intensity‐modulated 6 MV treatments 16 was improved and adapted for 15 MV (see Appendix 1). The algorithm calculated the whole‐body out‐of‐field dose of the coplanar treatments starting 4 cm longitudinal from the treatment volume (*∼*3 cm from the field edge). According to Kry et al.,^1^ the differences between treatment planning system (TPS) and measurements exceed 30% of the local dose as close as 3 cm from the field edge, and differences increase by orders of magnitude at greater distances. At 4 cm longitudinal from the treatment volume, the dose of the TPS (Varian Eclipse, AAA‐algorithm version 13.6.23) was fused with the model‐calculated 3D out‐of‐field dose resulting in a whole‐body photon dose [see Fig. 1(b)].

The peripheral neutron dose was calculated using an analytical model from the literature.^17^ This model was commissioned for TrueBeam linear accelerators (linacs) (Varian Medical Systems, Palo Alto, CA, USA), operated at 15 MV [see Fig. 1(c)]. The model assumed a point source of neutrons in the X‐ray producing target to predict the neutron fluence in the Alderson phantom. The fluence was converted to a neutron dose equivalent according to Sibert and Schumacher.^18^ Only the peripheral neutron dose was calculated since inside the primary X‐ray beam, the dose from neutrons can be neglected when compared to the photon dose.^12^, ^19^

#### 2.A.3 Whole‐body dose

To obtain a typical IGRT treatment dose, a daily CBCT 3D dose was added to the 3D photon dose per session. For the 15 MV treatments, the neutron dose equivalent per session was added. The voxel‐specific dose equivalents per session were multiplied with the number of sessions (see Table 1), resulting in the 3D dose equivalent per treatment [see Fig. 1(d)].

**Table 1:** The total treatment dose, total MUs, and MUs per treatment Gy. The treatments were planned by an experienced worker according to a strict protocol.^20^

Plan	Treatment dose ×fractions	Beam	Total MUs	MUs per treatment Gy
3DCRT	2.0 Gy *× *26 = 52.0 Gy	6 MV	7326 MUs	141 MUs/Gy
		15 MV	5846 MUs	112 MUs/Gy
IMRT	2.2 Gy *× *23 = 50.6 Gy	6 MV	22989 MUs	454 MUs/Gy
		15 MV	21661 MUs	428 MUs/Gy
VMAT	2.2 Gy *× *23 = 50.6 Gy	6 MV	13409 MUs	265 MUs/Gy
		15 MV	11847 MUs	234 MUs/Gy

### 2.B Whole‐body TLD measurements

The whole‐body photon and peripheral neutron dose measurements served as verification of the photon stray dose and neutron dose calculation.

LiF TLD‐chips (4.5 mm diameter, 0.6 mm thickness, Harshaw, Thermo Fisher Scientific, Waltham, MA, USA) were used to measure the in‐ and out‐of‐field dose of external therapy. For TLD100 (LiF:Mg,Ti) and TLD100H (LiF:Mg,Cu,P), the same thermal treatment, calibration procedure, and readout were used as described by Hauri and Schneider.^21^ The thermal treatment, calibration procedure, and readout for TLD600/700 (LiF:Mg,Ti) and TLD700H (LiF:Mg,Cu,P) were the same as applied to TLD100 and TLD100H, respectively. TLD100 contains the natural abundance of ^6^Li and ^7^Li, while TLD600 contains primarily ^6^Li. According to Schwahofer et al.,^22^ TLD600 and TLD100 show the same response to photon radiation since the number of neutrons in Li does not affect the energy bands of the TLD crystal. For the same reason, it was assumed in this manuscript that there is no difference in the response with photon radiation energy of TLD100H and TLD700H.

For each TLD, an individual photon dose‐to‐water calibration factor (in mGy*/*count) was determined using 6 MV nominal X‐ray energy applied with a TrueBeam linac. All absolute photon dose measurements were correlated to a Farmer Chamber 30013 (PTW, Freiburg, Germany). The irradiations and detector readouts were performed according to a strict protocol^21^ to ensure consistency of the measurements.

#### 2.B.1 Treatment intention, planning, and irradiation

The target volume of this study was a rhabdomyosarcoma in the prostate of an adolescent patient. The planning CT of the anthropomorphic Alderson phantom as well as the contouring of the target volume and organs were performed at one hospital.

The treatment planning of the 6 and 15 MV 3DCRT (four field box), IMRT (five fields with dynamic multileaf collimator (MLC)), and VMAT (one arc) treatments was done using the Eclipse TPS. All treatments were planned by an experienced worker. The 3DCRT treatments included a sequential boost and the intensity‐modulated treatments an integrated boost. The motivation regarding the treatment intention and the fractionation scheme, and a detailed description of the treatments and the strict planning guidelines can be found in Hälg et al.^20^

The diameter of the pelvis CBCT was 46.5 cm in a transversal slice and a field‐of‐view of *±*8.75 cm from the isocenter in the longitudinal direction. The CBCT protocol (version 2.5.28.0, half‐fan type, full trajectory, 125 kV_p_, 1080 mAs) was given by the vendor.

Using a conventional linac equipped with an on‐board imaging system (TrueBeam), the six treatments and the CBCT were irradiated onto the Alderson phantom, each time loaded with new TLDs. The phantom was positioned head first supine. For the 6 MV treatments and the CBCT scan, each measurement location in and on the phantom was equipped with a TLD100H stacked on top of a TLD100. For the 15 MV treatments, each measurement location was loaded with a TLD700H stacked on top of a TLD600. Confetti (made out of normal paper) were placed between all (TLD600, TLD700H)‐pairs to avoid an *α*‐particle contribution to the TLD700H signal originating from the ^6^Li(*n, α*) capture. The measurement locations were distributed in the Alderson phantom according to Hälg et al.^20^ Additionally, for the 15 MV treatments, the out‐of‐field photon dose of the skin was measured along a line from the pelvis to the nose of the phantom in steps of 10 cm. For this, the TLD700H were loaded in empty pill casings made of PMMA simulating the thickness of the skin. All absolute photon dose measurements were correlated to a Farmer Chamber 30013 since the chamber was used to determine the in‐field TLD‐calibration dose.

#### 2.B.2 Photon dose and mean photon energy of the CBCT and 6 MV treatments

The in‐ and out‐of‐field photon dose of the 6 MV treatments and the CBCT were measured separately using a combination of TLD100 and TLD100H chips. The two TLD types show a difference in response with photon radiation energy.^22^, ^23^ If calibrated with 6 MV nominal beam energy, TLD100 show an over‐ and TLD100H an under‐response toward lower energy (down to 0.1 MeV).^21^ By building the ratio of the TLD100 and the TLD100H measured doses, the mean photon energy can be determined. Using the photon energy at a specific measurement location, the TLD correction factors for the response with radiation energy can be determined. A comprehensive description of photon dose and the mean energy measurements for the CBCT and the 6 MV treatments is given in Hauri and Schneider.^21^ Furthermore, a detailed description of the uncertainties in photon dose and mean photon energy is presented.

#### 2.B.3 Photon dose of the 15 MV treatments

The whole‐body photon dose of the 15 MV treatments was determined for 189 locations in the Alderson phantom. The detected out‐of‐field photon dose by each TLD700H was corrected for the response with photon radiation energy. The individual correction factors were estimated by using the photon scatter contribution at each measurement location in the phantom.

The total out‐of‐field photon dose consists mainly of three contributions: patient scatter, collimator scatter, and head leakage.^8^, ^16^, ^24^ In the middle of a 30 *×* 30 *×* 30 cm^3^ water‐slab phantom (source surface distance = 85 cm), the mean energy of the three scatter contributions was measured using a combination of TLD700 and TLD700H.^21^ For a 10 *×* 10 cm^2^ field (defined by the MLC), the mean energy of patient scatter was measured at 15 cm distance to the field edge. For the same field size, the mean energy of collimator scatter was determined at 15 and 35 cm distance from the field edge. At the same locations, the mean energy of head leakage was measured for closed jaws and MLC. The separation of a field measurement into the three scatter contributions is described by Hauri et al.^16^

A previously developed 6 MV out‐of‐field dose model^16^ was improved and adapted for 15 MV (see Appendix 1). Using the adapted model, the doses of patient scatter, collimator scatter, and head leakage were calculated for each measurement location *l* in the phantom. The final out‐of‐field mean energy ${\bar{E}}_{l,c}^{\gamma }$ was determined by,

(1) $${\bar{E}}_{l,c}^{\gamma }=\frac{1}{\sum_{i}D_{i,l,c}^{\gamma }}\sum_{i}D_{i,l,c}^{\gamma }\cdot {\bar{E}}_{i,m}^{\gamma },$$

with *i *= {patient scatter, collimator scatter, head leakage}. $D_{i,l,c}^{\gamma }$ is the calculated dose at the measurement location *l* and $E_{i,m}^{\gamma },$ is the mean energy of the scatter contribution *i*.

The calculated out‐of‐field mean energies were used to determine the individual correction factors for the response with photon radiation energy of the TLDs. The multiplication of individual correction factors with the TLD700H‐detected dose resulted in the final photon dose.

As a consistency check, the TLD correction factors for the response with photon radiation energy were calculated for the 6 MV treatments (3DCRT, IMRT, and VMAT). In distinction to the 15 MV measurements, the photon energies for the 6 MV treatments were explicitly measured.^21^ Using Eq. (1), the mean photon energy was calculated for each measurement location of the 6 MV treatments. The calculated and measured mean energies were converted to correction factors for the TLD100H response with photon radiation energy. The calculated and measured correction factors for the 6 MV treatments were compared to estimate the uncertainty of the TLD700H photon dose measurement.

#### 2.B.4 Neutron dose equivalent of the 15 MV treatments

With a combination of TLD600 and TLD700H, the whole‐body neutron dose equivalent of the 15 MV treatments was determined. TLD700H is not affected by neutrons in the energy range of interest.^25^ TLD600 register photons and neutrons. Using the mean photon energy (Eq. (1)), the neutron signal detected by TLD600 in the phantom was corrected for the photon contamination measured by TLD700H. The measured neutron signal of a TLD600 was transformed to neutron dose equivalent (including fast neutrons) with a depth dependent conversion factor.^17^ Each TLD600‐specific depth in the phantom was calculated by using a straight line connecting the X‐ray producing target and the measurement location. The Alderson phantom was assumed to be a soft tissue‐equivalent (ICRU), with the exception of the lungs. For the lungs, a mass density of 0.25 *×*  *ρ* soft tissue was assumed (relative hydrogen content in lungs compared to soft tissue = 25% 26). For the 3DCRT and the IMRT treatments, the calculation of the depth in the phantom was straight‐forward since there was no gantry rotation during the beam‐on time. For the VMAT treatment, the control points of the one arc were grouped to six fields with different gantry angles and corresponding MUs per field. A more detailed description of the approximation of the VMAT plan by discrete fields can be found in Hauri et al.^16^

Compared to the TLD600‐registered signal from neutrons, the signal from photons is orders of magnitude higher in the target volume.^27^ Therefore, the measurement of neutron dose equivalents was only possible outside the treatment volume.

## 3 RESULTS

Unless otherwise stated, the mean and one standard deviation (*σ*) are presented. Consistent with the IAEA report,^28^ type A stands for the measured *σ* and the type B for the estimated *σ*.

### 3.A Whole‐body dose equivalent

#### 3.A.1 Photons

The deviation between the calculated whole‐body dose and the 183 point‐dose measurements of the CBCT scan was 0% *±* 14% (type A).

**Figure 2:**
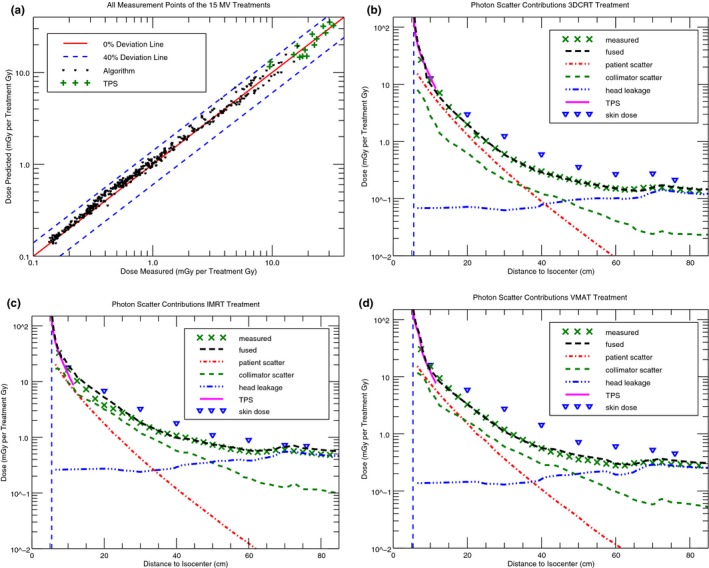
(a) The 438 out‐of‐field TLD700H photon dose point measurements in the Alderson phantom (head first supine) compared to the algorithm‐predicted doses for the three 15 MV treatments. Furthermore, the TPS‐calculated dose, the stray dose contributions, the total predicted doses fused with the TPS dose, and the measured doses along the MPAX for (b) the 3DCRT, (c) the IMRT, and (d) the VMAT treatment applied with a TrueBeam are shown. The vertical dashed lines in (b)–(d) represent the field edge calculated by the out‐of‐field dose algorithm. Additionally, the measured skin dose along a line from the pelvis to the nose of the Alderson phantom is plotted.

The measured mean photon energies of the three stray dose contributions can be seen in Table 2. Within the measurement uncertainties, there was no difference in the mean energies of the scatter contributions between the 15 and 6 MV field measurements. The measured mean energy of head leakage was the same at 15 and 35 cm distance from the field edge. For a nominal X‐ray energy of 15 MV, the mean energy of collimator scatter changes from *>*1.1 MeV at 15 cm to 0.5 MeV at 35 cm distance from the field edge. A similar change in the mean energy of collimator scatter was noticed for the 6 MV nominal X‐ray energy. However, close to the field edge, patient scatter is the dominating scatter contribution,^16^, ^24^ whereas the biggest contribution of collimator scatter relative to the other contributions is at around 35 cm distance from the field edge [see Figs. 2(b)–2(d)]. Hence, the mean energy of collimator scatter determined at 35 cm was used to calculate the final out‐of‐field mean energy for every measurement location [see Eq. (1)].

**Table 2:** The measured mean energies (${\overset{¯}{E}}_{i,m}^{\gamma }$) with the type B uncertainty ($\sigma /\sqrt{n}$ with n as the number of measurements) for *i *= patient scatter (*ps*), collimator scatter (*cs*), and head leakage (*hl*). The uncertainties were calculated according to Hauri and Schneider.^21^

Nominal X‐ray energy	${\overset{¯}{E}}_{i,m}^{\gamma }$ [MeV]		
	ps	cs	hl
6 MV	0.28 *± *0.03	0.62 *± *0.07	0.35 *± *0.03
15 MV	0.29 *± *0.03	0.53 *± *0.04	0.45 *± *0.04

The average mean photon energy measured for all out‐of‐field TLD locations and the three 6 MV treatments was (0.40 *±* 0.07) MeV (type A). For the same locations and treatments, an average mean photon energy of (0.40 *±* 0.03) MeV (type A) was calculated. For the 15 MV treatments, an average mean photon energy of (0.47 *±* 0.05) MeV (type A) was calculated. Hence, the TLD measurements of the 15 MV treatments needed similar corrections for the response with photon radiation energy as for the 6 MV treatments.^21^

For the 6 MV plans, the deviation between the calculated and measured correction factors for the TLD100 response with photon radiation energy was (0 *±* 1) % (type A). For TLD100H, the deviation between calculated and measured correction factors was (*−*1 *±* 1) % (type A). Using Gaussian error propagation, the uncertainty in the TLD700H dose measurement was determined to *±*2% (type B) (*σ *= *±*2% (type B) from the correction factor for the response with photon radiation energy and *σ *= *±*1% (type A) from the raw TLD700H dose measurement 21).

**Figure 3:**
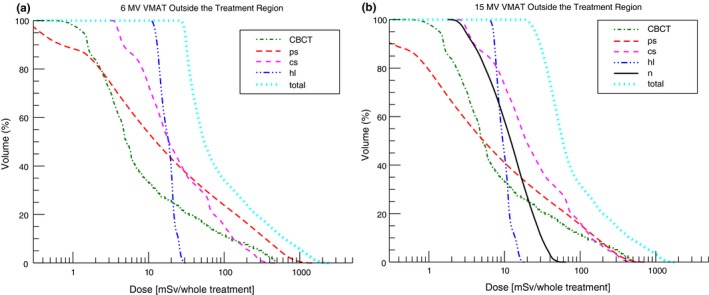
The out‐of‐field contributions (CBCT: 23 times a daily CBCT dose, ps: patient scatter, cs: collimator scatter, hl: head leakage, n: neurons dose equivalent) to the total DEVHs for the VMAT treatments applied with (a) 6 MV and (b) 15 MV nominal X‐ray energy.

In Fig. 3, the different out‐of‐field dose contributions for a VMAT treatment can be seen. For the 6 MV treatment, patient scatter was the largest out‐of‐field dose contribution. For 15 MV, collimator scatter was larger compared to patient scatter. Furthermore, the CBCT reached dose levels comparable to collimator scatter.

Averaged over all 15 MV treatments (3DCRT, IMRT, and VMAT), the deviation between the calculated whole‐body out‐of‐field photon dose and the measurement was (8 *±* 10) % (type A) [Fig 2 (a)]. For the 6 MV treatments, the deviation between calculated and measured out‐of‐field photon dose was (10 *±* 10) % (type A).

The photon dose calculated by the TPS was compared to the measurement in the transversal Alderson slices located 3 and 6 cm from the treatment volume. In the out‐of‐field region, the deviation between the TPS‐calculated and measured photon dose for the three 15 MV and three 6 MV treatments was (*−*5 *±* 21) % and (8 *±* 11) % (type A), respectively. For the same measurement locations, the deviation between the algorithm‐calculated and the measured photon dose was (*−*10 *±* 13) % and (*−*1 *±* 15) % (type A) for the 15 and 6 MV treatments, respectively. The agreement of the algorithm‐/TPS‐calculated dose and the TLD‐measured photon dose in the overlap region, justified the fusion of TPS and algorithm dose at 4 cm from the target volume. The fused photon dose compared to the measurement along the MPAX can be seen in Figs. 2(b)‐(d). In the same figures, the measured photon skin dose for the 15 MV treatments along a line from the pelvis to the nose of the phantom is plotted. For all treatments, the VMAT showed the largest and the IMRT the lowest skin dose relative to the measured dose along the MPAX. The improved stray dose algorithm underestimated the skin dose up to a factor of two.

#### 3.A.2 Neutrons

**Figure 4:**
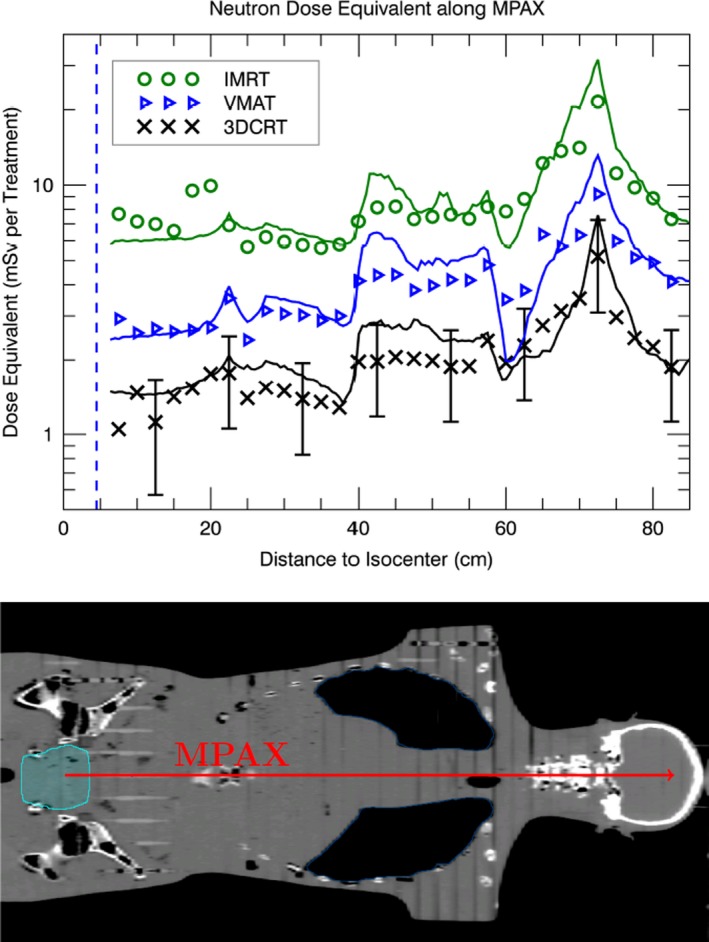
The measured (points) and calculated (solid lines) neutron dose equivalent for the 3DCRT, IMRT, and VMAT treatments along the MPAX. For the measurements, a TrueBeam linac operated at 15 MV nominal X‐ray energy was used. The 95% confidence interval for the measured neutron dose equivalent (indicated by the error bars) decreased rapidly from *±*100% to *±*40% (type B) with increasing distance to the central field axis. The dashed line indicates the edge of the target volume. Furthermore, the Alderson phantom with the outline of the target volume (rhabdomyosarcoma) in the hip region can be seen. The arrow represents the MPAX and is scaled to the upper figure.

In Fig. 4, the measured and calculated neutron dose equivalent along the MPAX can be seen. The calculation and measurements were in good agreement. The large photon signal relative to the neutron signal close to the target volume resulted in a large uncertainty in the neutron dose equivalent. However, the uncertainty resulting from the photon contamination was below *±*10% (type B) at locations *≥*3 cm from the target volume. The overall uncertainty in the measured neutron dose equivalent was below *±*30% (type B) at locations *≥*3 cm from the target volume. The difference between the calculated and measured neutron dose equivalent was 18 *±* 27% (type A) for the three 15 MV treatments. Hence, the calculation and the measurement were in agreement within the measurement uncertainties.

The calculated and measured neutron dose equivalent showed a strong dependence with depth. There was no significant difference (*α < *0.05) in the measured neutron dose equivalent per MU along the MPAX between the 3DCRT, IMRT, and VMAT treatment. However, for measurement points close to the surface, the treatments showed significant differences in neutron dose equivalent. The neutron dose close to the surface was dependent on the gantry angles of the treatment fields. The neutron model overestimated the dose equivalent in the lungs.

#### 3.A.3 Total dose equivalent

**Figure 5:**
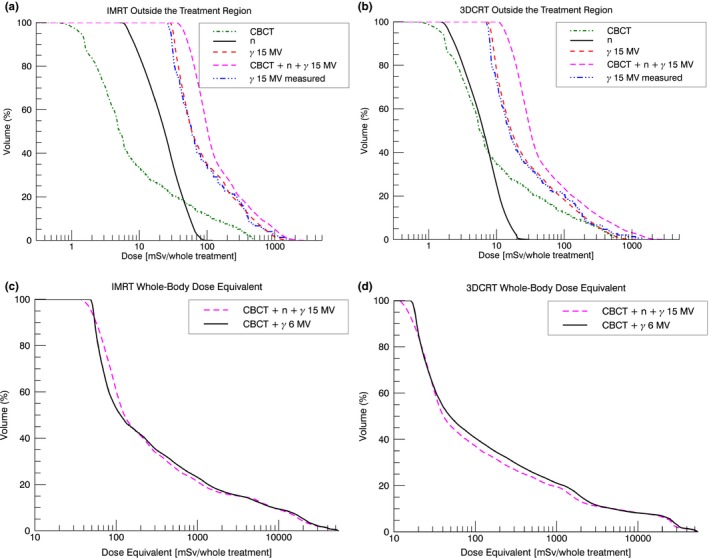
Out‐of‐field DEVHs of the various contributions (CBCT: daily CBCT dose *×* number of treatment sessions, n: neutron dose equivalent, *γ*: photon dose) and the total dose for the 15 MV (a) IMRT and (b) 3DCRT plan per whole treatment. “*γ* 15 MV” indicates the calculated whole‐body photon dose. “*γ* 15 MV measured” was determined from the 151 out‐of‐field measurement locations distributed in the Alderson phantom. In (c) for IMRT and (d) for 3DCRT, the in‐ and out‐of‐field whole‐body DEVHs for the 6 MV and the 15 MV treatments are plotted.

In Figs. 5(a) and 5(b), the out‐of‐field contributions to the total dose equivalent volume histograms (DEVHs) of the IMRT and the 3DCRT 15 MV treatments are plotted (on a logarithmic scale). There was good agreement between the photon DEVH determined by the 151 out‐of‐field measurement points and DEVH determined by the calculated photon dose. The DEVHs of the VMAT treatments (not plotted) were between the DEVHs of the 3DCRT and the IMRT treatments. For all energies and treatment techniques, the photon stray dose caused by the primary beam was the biggest contribution to the total out‐of‐field dose. At the edge of the CBCT field‐of‐view, the daily CBCT dose reached levels comparable to the 3DCRT out‐of‐field dose [Fig. 5(b)]. In Figs. 5(c) and 5(d), the whole‐body DEVH of the 6 and 15 MV treatments can be seen. For the same treatment technique, the DEVHs showed only small differences between the two beam energies.

Outside the target volume, the deviation between the calculated and measured final dose equivalent was (10 *±* 11) % and (8 *±* 8) % (type A) for the 15 MV and the 6 MV treatments, respectively. The measured uncertainty in the final dose equivalent was similar to the uncertainty in the photon scatter dose since the stray dose was the biggest contribution of the total dose equivalent.

**Figure 6:**
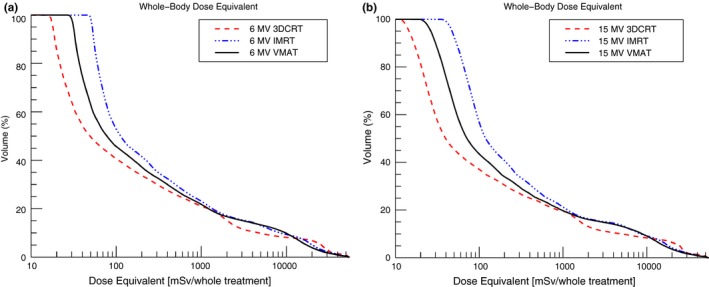
The whole‐body DEVHs for (a) the 6 MV (b) the 15 MV treatments.

In Fig. 6, the whole‐body DEVHs of the different treatment techniques are shown. In the high and intermediate dose region (*>*3 Sv or 5% of the prescribed dose), the DEVHs for the intensity‐modulated treatments were similar. In the same dose region, the DEVHs of the 3DCRT treatments were different from the intensity‐modulated treatments. For the same beam energy, the minimum dose of the 3DCRT treatment was two times lower than for the VMAT treatment and four times lower than for the IMRT treatment.

## 4 DISCUSSION

Averaged over all treatment techniques and nominal X‐ray energies, the calculated whole‐body dose equivalent agreed within (9 *±* 10) % (type A) compared to the measured dose equivalent. This agreement was sufficient to determine differences in the whole‐body dose between the investigated treatments.

### 4.A Photon dose

The small deviation between the predicted and measured CBCT dose justified the presented method to calculate the whole‐body imaging dose.

Close to the target volume, the CBCT dose was a substantial contribution to the out‐of‐field dose resulting from a treatment (Fig. 5). In the field‐of‐view of the CBCT, the dose was almost constant.^29^ Furthermore, the field‐of‐view extended around 4 cm over the border of the target volume. In this area, the dose caused by scatter radiation of the primary beam dropped rapidly with increasing distance to the target volume. The CBCT dose decreased exponentially with increasing distance to the field‐of‐view. Hence, a smaller field‐of‐view is beneficial regarding radiation protection of the patient. The contribution of imaging to the total dose equivalent was similar for all investigated treatments since the number of sessions and the fraction doses were comparable for all treatments (see Table 1). It follows that the results obtained in this work are valid even if the imaging dose is excluded from the comparisons. A detailed discussion of the CBCT dose in context of the treatment dose can be found in the literature.^14^

The mean photon energies calculated/measured outside the treatment volume were in agreement with reported out‐of‐field mean energies for 6 and 15 MV (static fields defined by the MLC or IMRT fields).^30^, ^31^, ^32^ Kry et al.^30^ simulated the same average photon energy (0.4 MeV) for a 6 MV field (defined by the MLC) as we calculated/measured in the current work. Using the simulated average mean energy, they applied an overall correction factor for TLD100 to correct the out‐of‐field dose measurements. However, a general correction factor leads to a systematic error in dose.^21^, ^31^ Using the presented method to calculate the mean photon energy, this systematic error in the dose correction can be avoided.

The spectrum of patient scatter for a Simens Primus 6/15 linac was MC simulated by Chofor et al.^33^ A similar photon energy of patient scatter for 6 and 15 MV beams was reported. This is in agreement with our work. Photons of lower energies have an increased probability of large Compton scatter angles compared to high‐energy photons. Hence, particularly photons of lower energies from the primary X‐ray spectrum cause patient scatter. The fluence ratio of low‐energy photons to high‐energy photons is larger for 6 MV than for 15 MV beams. This could be one of the reasons why patient scatter dose was higher for 6 MV than for 15 MV treatments.

Compared to the primary beam, the X‐ray spectrum in the peripheral region is softer such that an increase in organ‐specific relative biological effectiveness (RBE) for carcinogenesis is expected.^34^ With the presented method, the dose and corresponding mean photon energy can be calculated separately for patient scatter, collimator scatter, and head leakage. Hence, for every scatter contribution, a separate RBE for cancer induction can be determined.

Close to the field edge, where patient scatter and collimator scatter dominated (see Fig. 2), the 6 MV treatments showed a higher dose than the 15 MV counterparts. This is in agreement with a MC study from the literature.^35^ For a standard field, patient scatter was increased by a factor of two for 6 MV compared to 15 MV (Fig. 8), whereas collimator scatter was reduced just by a factor of 1.5 for 6 MV compared to 15 MV (Fig. 9). This factor was reduced further because collimator scatter scales with the applied MUs^16^ and the 6 MV treatments needed more MUs compared to the 15 MV treatments (Table 1).

Ruben et al.^8^, ^24^ measured the components of the out‐of‐field dose for IMRT and 3DCRT fields up to a distance 40 cm from the isocenter. In agreement with our work, they reported a decreasing patient scatter dose for increasing nominal X‐ray energy. Furthermore, they measured nearly the same 3DCRT out‐of‐field dose for 6 and 15 MV treatments. In their study, both 3DCRT treatments utilized the same MUs. We noticed an overall lower photon dose with the 15 MV than with the 6 MV 3DCRT treatment. In our study, lesser MUs were needed to apply the 15 MV than the 6 MV treatments.

The improved general model for stray dose calculation^16^ predicted the measured off‐axis photon dose contribution in the anthropomorphic phantom well. To our knowledge, this is the only analytical model for whole‐body photon dose prediction for static and intensity‐modulated treatments. An analytical model to calculate the out‐of‐field dose for intensity‐modulated treatments was introduced by Sanchez et al.^32^ Their model is only applicable for distances *≥*10 cm from the field edge because they neglected the patient scatter contribution. However, close to the field edge, the largest out‐of‐field dose gradients are present. Furthermore, the TPS cannot be used to calculate the out‐of‐field dose up to 10 cm from the field edge since differences between TPS and measurements exceed 30% of the local dose as close as 3 cm from the field edge,^1^ and differences increase by orders of magnitude at greater distances.

An aspect of neutron interaction in the phantom is the production of capture gamma‐ray emission. The dose contribution from this emission can be neglected for photon radiotherapy since it is small compared to the scatter photon dose of the primary beam.^19^ However, using TLD700H the capture gamma rays were measured together with the scatter photon dose. Furthermore, the capture gamma‐ray contribution was included in the predicted head leakage dose since the scatter dose model was adjusted using ionization chamber measurements (see Appendix 1). The TLD700H measurements agreed well with the calculated stray dose in the off‐axis region, where head leakage was dominating [see Figs. 2(b)–2(d)].

### 4.B Neutron dose equivalent

Compared to the measurements, the model overestimated the neutron dose in the phantom in average by 18%. The overestimation was larger for the 3DCRT compared to the IMRT treatment. The 3DCRT treatments showed an average field opening of around 9 *×* 10 cm^2^ compared to an opening of 3 *×* 10 cm^2^ for the IMRT treatments. Howell et al.^36^ measured less neutron fluence per MU for a 3DCRT than for an IMRT treatment. The MLC are an additional source of photoneutron production.^1^ The model was commissioned for closed jaws and MLC and therefore, it predicts a conservative estimate of the neutron dose equivalent for a patient treatment.

Within the uncertainties, the prediction by the neutron dose model was in agreement with the measurements. Along the MPAX, the neutron dose peaked in the neck of the Alderson phantom for the calculation as well for the measurement (Fig. 4). The reason for this is that in the neck region, the neutrons penetrated the smallest amount of tissue to reach the MPAX compared to other parts of the phantom. The largest overestimation in neutron dose by the model compared to the measurements was seen in the lungs. A simple scaling with the density of the lungs was used to calculate the depth in the anthropomorphic phantom. A wrongly assumed density for lung tissue can explain the overestimation in neutron dose.

H¨alg et al.^12^ measured the neutron dose equivalent using track etch detectors for the same treatment intention and techniques as presented in the current work. For all treatments, the detectors showed a systematic three times reduced neutron dose equivalent per MU compared to the results presented here. Along the MPAX, there was no significant difference in the neutron dose equivalent per MU between the three different techniques. Hence, the neutron dose equivalent inside the phantom scaled with the applied MUs independently of the treatment technique. This was not true for the neutron dose per MU closer to the phantom surface. Close to the surface, the neutron dose showed a significant difference between the three 15 MV treatment techniques (3DCRT, IMRT, VMAT). Neutron dose close to the surface was strongly dependent on gantry angle of the treatment fields.

### 4.C Total dose equivalent

The DEVHs of the calculated photon stray dose were in good agreement with the DEVHs of the measurement (Fig. 5). Hence, the measurement locations represented a whole‐body photon dose well. In comparison, DEVH of the calculated neutron dose equivalents showed more dose per volume than the DEVHs from the measurements. This can be explained by the fact that most of the TLD measurement locations were deeper than 1 cm in the phantom.^20^ The locations were chosen such that they cover all ICRP‐recommended organs.^37^ The neutron dose decreases rapidly with increasing depth in the phantom [Fig. 1(a)]. In terms of radiation protection, the high neutron dose contribution down to 1 cm in the patient is of less importance since most ICRP organs are located deeper in the body.

For high and intermediate doses (*>*3 Gy or 5% of the prescribed dose), the DEVHs for the same technique were similar for the two nominal X‐ray energies [Figs. 5(a) and 5(b)].

**Figure 7:**
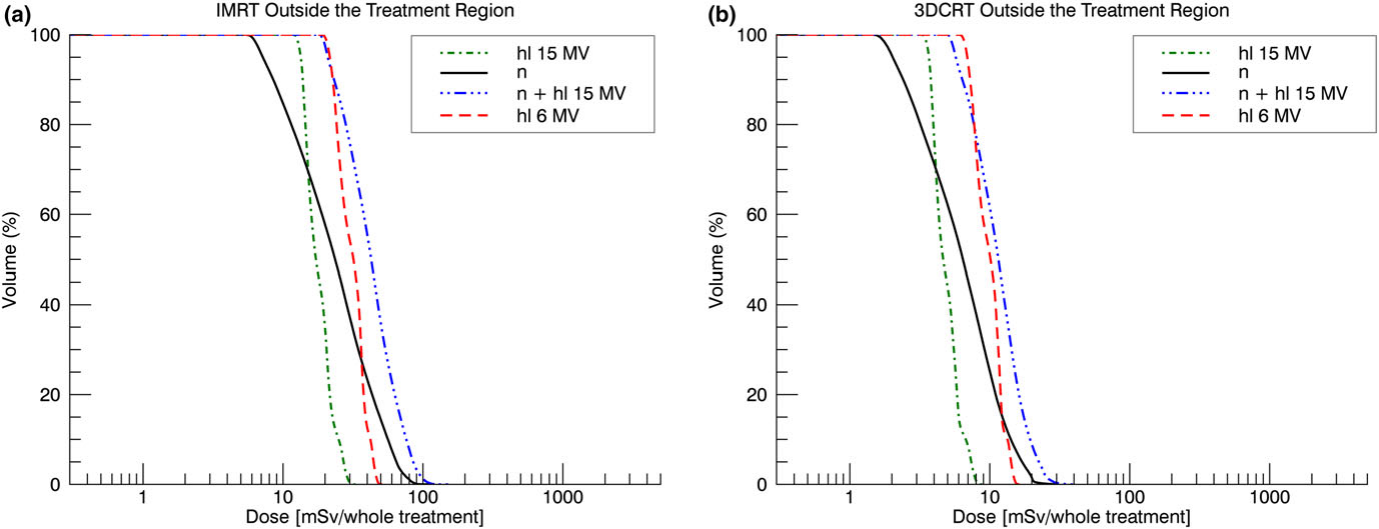
Head leakage (hl) and neutron (n) DEVH for (a) the IMRT and (b) the 3DCRT treatments.

**Figure 8:**
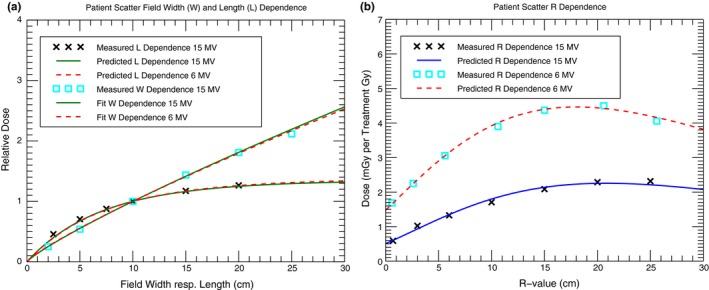
In (a) the measured and model‐predicted field length dependence of patient scatter. Furthermore, the fit of the field width dependence. In (b) the measured and model‐predicted dose along a line parallel to the central field axis 15 cm from the field edge (R‐dependence 16).

The out‐of‐field dose close to the target volume was higher for 6 MV than for 15 MV. Patient scatter and to a lesser extend collimator scatter are the dominating out‐of‐field dose contributions close to the target volume. 6 MV nominal X‐ray energy showed a two times increased patient scatter and a similar collimator scatter contribution compared to 15 MV (see Appendix 1). This explained the larger body volume receiving doses of 0.2–3 Sv for 6 MV compared to 15 MV [Figs. 5(c) and 5(d)]. At around 50 cm from the isocenter, head leakage and neutrons were the dominating out‐of‐field dose contributions [Figs. 2(b)–2(d)]. Hence, for doses *<*0.2 Sv, the differences in the DEVHs were dependent on the difference between head leakage of 6 MV compared to head leakage and neutron dose of 15 MV. Head leakage and neutron dose scale linearly with MU and are in a first approximation independent of the field shape.^1^ The relative difference in MUs for the 6 MV compared to the 15 MV treatment was smaller for the intensity‐modulated treatments when compared to the 3DCRT treatments (see Table 1). This explained the crossing of the 6 and 15 MV DEVHs for the intensity‐modulated treatments at 0.2 Sv (see Fig. 5c). For the 3DCRT treatments, the DEVH of the 15 MV plan was equal or below the 6 MV DEVH. Hence, regarding radiation protection of the patient, the 3DCRT 15 MV treatment was superior compared to the 6 MV treatment. Head leakage and neutron DEVHs can be seen in Fig. 7.

**Figure 9:**
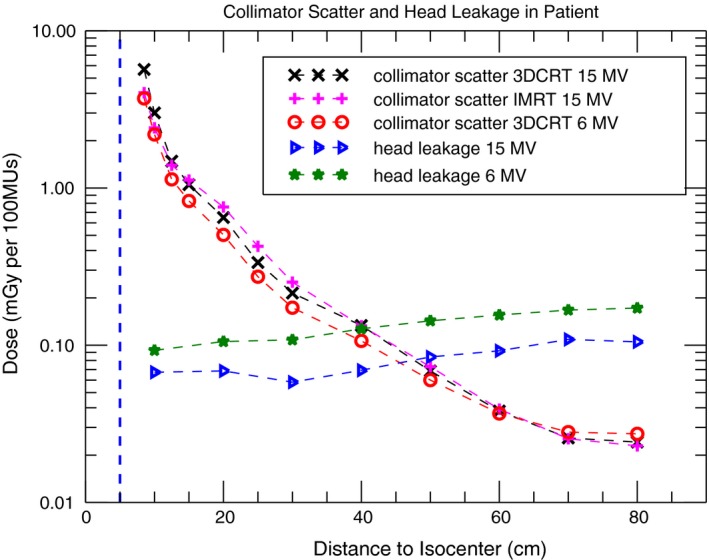
Collimator scatter and head leakage for two nominal X‐ray energies along the MPAX for a constant patient thickness of 10 cm. For 15 MV, collimator scatter is shown for a static IMRT and a 3DCRT field.

Head leakage was almost constant in the phantom, whereas neutron dose was inhomogeneous. For all techniques, the minimum dose was higher for 6 MV than for 15 MV. This was caused by a smaller leakage dose for 15 MV than for 6 MV (Fig. 9) and the low neutron dose in the center of the body [Fig. 1 (c)]. Head leakage is assumed to be reduced because of more forward‐directed photons in the X‐ray producing target for 15 MV than for 6 MV.

Between 15 and 6 MV, the dose equivalent was similar for the same treatment technique such that the resulting cancer risk might not be clinically observable. Independent of the nominal X‐ray energy, the dose equivalent in the low and intermediate dose region was increased by a factor of two for the VMAT compared to the 3DCRT treatment and by a factor of four for the IMRT compared to the 3DCRT treatments. Using VMAT, similar dose distributions in the target volume can be achieved as for IMRT.^38^ However, the out‐of‐field dose is different between the two techniques, favoring VMAT for radiation protection of the patient. This is because usually a shorter beam‐on time is needed for VMAT compared to IMRT and collimator scatter, head leakage, and neutron dose are linearly scaling with the applied MUs. Varian linacs have been shown to produce the most photoneutrons compared to other vendors.^1^ This has raised concerns about radiation protection of the patient when treating with Varian linacs operated at nominal X‐ray energies higher than 10 MV.^7^, ^39^ Far from the treatment region (*>*50 cm), the photoneutron dose equivalent was similar to head leakage. Neutron dose and head leakage scaled with the applied MUs. Varian accelerators (600C, 21‐iX) show a reduced leakage dose compared to other vendors (Elekta Synergy‐II, Siemens Primus).^40^ Hence, regarding radiation protection of the patient for high‐energy treatments, the choice of treatment machine should not only be based on neutron production but rather on the total dose equivalent including photon scatter.

Multiple studies reported an increased cancer risk based on an increased dose equivalent for high energy compared to low‐energy radiotherapy.^35^, ^39^ The increased dose equivalent for high compared to low‐energy X‐ray therapy was reported to be caused by the additional neutron dose. However, the neutron energies in these publications were overestimated resulting in an overestimation of neutron dose. An extensive discussion of the overestimation in neutron dose reported by the literature can be found in Kry et al.^7^ For the same treatment technique, we did not notice an increase in the whole‐body dose equivalent for increasing nominal X‐ray energy (up to 15 MV). For IMRT treatments, the findings are in agreement with an MC study made by Kry et al.^7^ They found that, the calculated 6 and 18 MV out‐of‐field doses were similar for IMRT.

A shortcoming of this study is that the investigation was focused only to one treatment location (rhabdomyosarcoma in the prostate). In addition, the whole‐body doses were calculated using an anthropomorphic phantom and not a patient CT. This had the advantage that the calculation could be directly compared to the measurements. Nevertheless, the dose models used in this manuscript are applicable to other treatment locations and patient geometries. The photon dose calculated using the stray dose model^16^ was in good agreement (absolute mean deviation of 22%) with whole‐body dose measurements of 6 MV treatments for Hodgkin disease (involved field and involved node), and for treatments of an ependymoma in the head (3DCRT, IMRT, and VMAT).^41^ Regarding out‐of‐field dose, further investigations are planned for different treatment indications. These investigations are urgently needed.^5^

The calculated CBCT dose distribution was based on whole‐body measurement. It is time consuming and not practical to measure the dose of various CBCT protocols. Furthermore, the choice of the protocol influences the dose distribution in the field‐of‐view region.^29^ Analytical models to calculate the CBCT dose are available but they lack the ability to calculate the dose outside the field‐of‐view.^42^

We did not include the out‐of‐field dose caused by electron contamination. Outside the primary beam, the dose close to the surface can be increased by a factor of 4 compared to inside the body (*>*2 cm).^10^ However, most critical organs are located in a patient depth outside the reach of these electrons. Furthermore, usually treatments are applied using multiple gantry angles, which reduces the increased surface dose caused by electron contamination compared to the dose in larger depths [see Figs. 2(b)–2(d)]. Nevertheless, the skin dose was underestimated by the photon dose algorithm used in this work.

For the calculation of the total whole‐body dose of a real patient, the planning CT can be fused with a phantom containing the contours of critical tissues.^43^ Such a feature is not yet clinically available. Using the application programming interface of the Eclipse TPS, it is planned to fuse the limited patient CT with a computational human phantom from a library to generate a whole‐body representation of the patient.

## 5 CONCLUSION

The calculated whole‐body dose equivalent for IGRT treatments helped to understand the importance of the scatter contributions in different areas of the patient body. The calculations agreed well with measurements and reported values from the literature. For intensity‐modulated treatments, VMAT should be used instead of IMRT because of its shorter beam‐on time, which reduces the out‐of‐field dose.

One of the novelties in this paper was the analytically calculated mean photon energy for every point in the patient outside the primary beam. The calculated energies agreed with measurements and reported values from the literature. The mean photon energy can be used to correct for a variation in response of a detector and to estimate the RBE for the scatter contributions.

The neutron dose calculated by the model overestimated the neutron dose equivalent by around 20% compared to the measurements. The relatively small error in photon dose calculations compared to the relative large error in neutron dose equivalent reduced the overall error to around *±*20% (type B) since the photon dose was the main contributor to the out‐of‐field dose.

Second cancer risk estimations are limited by the errors in the risk model and the whole‐body dose calculation. In this work, the accuracy and precision of the dose estimation were improved. Further research should be carried out to improve cancer risk models and whole‐body dose calculations to achieve better estimates in second cancer induction.

## CONFLICT OF INTEREST

The authors have no relevant conflicts of interest to disclose.

## APPENDIX 1

### 6 MV and 15 MV PHOTON SCATTER MODELS

A general model for photon stray dose calculation developed for a TrueBeam operated at nominal X‐ray energy of 6 MV^16^ was improved and adjusted for 15 MV. The total absorbed dose outside the treatment field *D*_*t*_ can be described as the sum of three contributions,

(2) $$D_{t}=D_{\mathrm{ps}}+D_{\mathrm{cs}}+D_{\mathrm{hl}}$$

Patient scatter *D*_*ps*_ is the dose mainly produced by Compton scatter photons of the treatment field penetrating the patient. Photons of the primary field scattered at the jaws and MLC are described by collimator scatter *D*_*cs*_. Head leakage is the out‐of‐field dose contribution from photons which originate from the X‐ray producing target and leaking through the gantry head shielding *D*_*hl*_.

#### Patient scatter model

The four parameters of the mechanistic model of patient scatter see [Eq. (8) from Hauri et al.^16^] were adapted for 15 MV nominal X‐ray energy. The total absorbed dose was measured in simple geometries using a Rigid Stem ionization chamber 30016 (PTW, Freiburg, Germany). Patient scatter was extracted from the total out‐of‐field measurement and the four physically motivated fit parameters were determined. In Table 3, the evaluated parameters for 15 MV nominal X‐ray energy can be seen. For comparison, the parameters for 6 MV are shown, too. The field width and attenuation coefficient [*C*_*W*_ and $\mu_{\mathrm{ps}}^{\gamma }$)] showed almost no difference between the two nominal X‐ray energies. However, the backscatter contribution constant *C*_*B*_ showed a clear decrease for 15 MV compared to 6 MV. Furthermore, the normalization constant *C*_*N*_ describing the magnitude of patient scatter decreased by a factor of 1.8 for 15 MV compared to 6 MV.

**Table 3:** The four measured parameters for the patient scatter model [Eq. (8) from Hauri et al.^16^] and the attenuation of the primary beam (pb)

Beam	*C*_*W*_	*C*_*N*_	*C*_*B*_	$\mu (E_{\mathrm{ps}}^{\gamma })$	$\mu (E_{pb,10\times 10cm^{2}}^{\gamma })$ 44
6 MV	0.845	0.724 mGy/Gy	0.203	0.007 22 mm^*−*1^	0.005 41 mm^*−*1^
15 MV	0.857	0.400 mGy/Gy	0.174	0.007 68 mm^*−*1^	0.004 10 mm^*−*1^

In Fig. 8, we see the comparison between patient scatter resulting from 15 to 6 MV nominal X‐ray energy. The normalized patient scatter as a function of field widths and lengths showed no difference between 15 and 6 MV [Fig. 8(a)]. The absolute patient scatter dose 15 cm from the field edge along a line parallel to the central field axis (R‐dependence 16) showed a higher dose for 6 MV than for 15 MV [Fig. 8(b)].

For 6 MV, Chofor et al.^33^ simulated an almost twofold increase in patient scatter compared to 15 MV. Ruben et al.^8^ measured a 1/3 higher patient scatter dose for 6 MV than for 18 MV. However, the difference in patient scatter between two nominal X‐ray energies is dependent on the location in the patient, as can be seen in Fig. 8(b). Patient scatter is independent from the beam head design and is an unavoidable result of external radiotherapy.^33^ Table III shows that primarily the normalization constant of the patient scatter model has to be adjusted when using different beam energies. The normalization constant can be determined by one measurement set‐up. However, for flattening filter free beams, all model parameters could be different compared to flattening filter beams.

#### Collimator scatter and head leakage models

The empirical models of collimator scatter and head leakage^16^ were improved. For 6 and 15 MV nominal X‐ray energy, collimator scatter and head leakage were measured with a Rigid Stem ionization chamber placed in a 30 *×* 30 *×* 30 cm^3^ RW3 phantom. For the measurement, the phantom was moved along the MPAX. All measurements of collimator scatter and head leakage are discussed for locations in the direction “isocenter towards gun” and collimator rotation of 0^*◦*^.

For each energy, the measurements were made at different depths in the RW3 phantom (7 cm, 15 cm, 23 cm) with and without a lateral displacement of 8 cm from the MPAX. This resulted in nine dose curves parallel to the MPAX, assuming lateral symmetry for collimator scatter and head leakage. Collimator scatter was measured for different symmetric field sizes defined by the MLC. Head leakage was measured by closing the jaws and MLC. The dose between the measurement locations along the MPAX was calculated by a linear interpolation. To evaluate the exponential decrease with depth, depth dose curves of the collimator scatter and head leakage were measured at different distances along the MPAX.

For fixed jaw positions, we noticed a change in collimator scatter with a changing MLC width opening. To account for this effect, collimator scatter for different MLC width openings (jaws constant at 10 *×* 10 cm^2^) was measured at different distances along the MPAX. With the results, a scaling matrix was calculated which corrected for the change of collimator scatter with changing MLC width opening. No such effect was noticed for a variation in the MLC length opening.^16^

To calculate collimator scatter and head leakage for a treatment, the dose contributions along the MPAX were scaled with the exponential decrease in depth. To determine the depth, the patient was assumed to be water equivalent with the exception of the lungs (*ρ*_lungs_ = 0.25 *×* *ρ*_water_). A more detailed description how the depth in the patient was calculated for each treatment field can be found in Hauri et al.^16^

In Fig. 9, we see collimator scatter and head leakage per 100 MUs along the MPAX in the center of the RW3 phantom. Collimator scatter is shown for a fixed jaw field size of 10 *×* 10 cm^2^ in combination with an MLC field size of 9 *×* 10 cm^2^, representing a 3DCRT field, and for an MLC field size of 3 *×* 10 cm^2^, representing an IMRT field. The field sizes were similar to the field sizes of the 3DCRT and IMRT treatments measured in the presented work. For calculation of the dose, the IMRT treatment with dynamic MLC was approximated by static fields. For the 3DCRT fields, the ratio of collimator scatter between 15 and 6 MV decreased from 1.5 to 0.88 for increasing distance to the isocenter. This is in agreement with other publications, reporting increased collimator scatter close to the field edge with increasing energy.^1^

Close to the field edge, collimator scatter of the 15 MV IMRT field was 0.7 times smaller than the 15 MV 3DCRT field. We believe that this difference was caused by the shielding effect of the MLC for photons of the primary beam scattered in the gantry head. The ratio of collimator scatter between the IMRT and the 3DCRT field rose to 1.3 (25 cm from isocenter) with increasing distance to the field edge. As a potential explanation serves the additional photon scatter from the MLC, which outweighed the shielding effect of the MLC. At distances *≥*40 cm from the isocenter, there was no difference in collimator scatter per MU between the 3DCRT and IMRT field.

Along the MPAX, head leakage for 6 MV was around 1.6 times higher than head leakage for 15 MV. For both nominal X‐ray energies, head leakage showed the same increase for increasing distance to the isocenter. A possible explanation could be the change in photon attenuation for different paths through the gantry head's shielding.

In Table 4, we see the measured attenuation coefficient for collimator scatter and head leakage. In contrast to the expectation, the attenuation of the scatter contribution was higher for 15 MV than for 6 MV. The attenuation for collimator scatter was calculated using a straight line connecting the rear jaw and the point of interest in the patient. For head leakage, the attenuation was calculated using a straight line connecting the X‐ray producing target with the point of interest located in the patient.

**Table 4:** The attenuation coefficients in water for collimator scatter and head leakage

Beam	$\mu {\left(E_{\mathrm{cs}}^{\gamma }\right)}_{m}$	$\mu {\left(E_{\mathrm{hl}}^{\gamma }\right)}_{m}$
6 MV	0.001 96 mm^−1^	0.002 70 mm^−1^
15 MV	0.003 05 mm^−1^	0.003 31 mm^−1^
